# Supplementary material for: Metacognition across domains: Is the association between arithmetic and metacognitive monitoring domain-specific?
Source: PLoS One. 2020 Mar 12;15(3):e0229932. doi: 10.1371/journal.pone.0229932 (PMC7067420; doi:10.1371/journal.pone.0229932)
Supplement: S1 Appendix — (DOCX) [file pone.0229932.s001.docx]

# S1 Appendix A.

## Descriptive statistics.

**Table A1. Descriptive statistics of the key variables in 8-9-year olds (Grade 3; Study 1).**

|  | *n* | *M* | *SD* | Range |
| --- | --- | --- | --- | --- |
| **Arithmetic** |  |  |  |  |
| *Custom task* |  |  |  |  |
| Accuracy | 141 | 0.94 | 0.06 | [0.75-1.00] |
| Response time (ms) | 141 | 3833 | 1479 | [1537-8543] |
| *Standardized task* |  |  |  |  |
| Total score | 144 | 77.70 | 19.20 | [36.00-133.00] |
| **Spelling** |  |  |  |  |
| *Custom task* |  |  |  |  |
| Accuracy | 145 | 0.78 | 0.12 | [0.45-0.98] |
| Response time (ms) | 145 | 2434 | 729 | [1188-4586] |
| *Standardized task* |  |  |  |  |
| Total score | 144 | 30.00 | 8.28 | [9.00-43.00] |
| **Metacognitive monitoring** |  |  |  |  |
| *Calibration of confidence* |  |  |  |  |
| In arithmetic task | 141 | 1.88 | 0.12 | [1.53-2.00] |
| In spelling task | 145 | 1.53 | 0.25 | [0.88-1.97] |
| **Control** |  |  |  |  |
| *Intellectual ability* |  |  |  |  |
| Raven | 143 | 36.10 | 7.43 | [13.00-52.00] |
| *Motor Speed* |  |  |  |  |
| Accuracy | 143 | 0.98 | 0.03 | [0.90-1.00] |
| Response time (ms) | 143 | 593 | 135 | [390-1044] |

**Table A2. Descriptive statistics of the key variables in 7-8-year-olds (Grade 2; Study 2).**

|  | *n* | *M* | *SD* | Range |
| --- | --- | --- | --- | --- |
| **Arithmetic** |  |  |  |  |
| *Custom task* |  |  |  |  |
| Accuracy | 73 | 0.89 | 0.12 | [0.50-1.00] |
| Response time (ms) | 73 | 4384.30 | 1541.92 | [1606.13-8273.17] |
| *Standardized task* |  |  |  |  |
| Total score | 68 | 27.24 | 6.74 | [14-43] |
| **Spelling** |  |  |  |  |
| *Custom task* |  |  |  |  |
| Accuracy | 76 | 0.70 | 0.13 | [0.43-1.00] |
| Response time (ms) | 76 | 2994.69 | 1063.48 | [1052.67-7368.39] |
| *Standardized task* |  |  |  |  |
| Total score | 68 | 32.65 | 5.66 | [17-42] |
| **Metacognitive monitoring** |  |  |  |  |
| *Calibration of confidence* |  |  |  |  |
| In arithmetic task | 73 | 1.74 | 0.25 | [0.97-2.00] |
| In spelling task | 76 | 1.38 | 0.26 | [0.97-1.93] |
| **Control** |  |  |  |  |
| *Intellectual ability* |  |  |  |  |
| Raven | 68 | 29.16 | 8.39 | [11-48] |
| *Motor Speed* |  |  |  |  |
| Accuracy | 74 | 0.99 | 0.03 | [0.95-1.00] |
| Response time (ms) | 74 | 634.68 | 138.56 | [413.63-1073.11] |
