# Supplementary material for: Metacognition across domains: Is the association between arithmetic and metacognitive monitoring domain-specific?
Source: PLoS One. 2020 Mar 12;15(3):e0229932. doi: 10.1371/journal.pone.0229932 (PMC7067420; doi:10.1371/journal.pone.0229932)
Supplement: S2 Appendix — (DOCX) [file pone.0229932.s002.docx]

# S2 Appendix B.

## All intercorrelations.

**Table B1. Correlational analyses between the administered measures in 8-9-year-olds (Grade 3; Study 1).**

|  |  |  | 1a | 1b | 1c | 2a | 2b | 2c | 3a | 3b | 4 |
| --- | --- | --- | --- | --- | --- | --- | --- | --- | --- | --- | --- |
| **Arithmetic** | Custom task | 1a. Arithmetic Acc | - |  |  |  |  |  |  |  |  |
|  |  | 1b. Arithmetic RT |  |  |  |  |  |  |  |  |  |
|  |  | *r* | .12 |  |  |  |  |  |  |  |  |
|  |  | *p* | .14 | - |  |  |  |  |  |  |  |
|  |  | BF_10_ | 0.31 | - |  |  |  |  |  |  |  |
|  | Standardized task | 1c. TTA |  |  |  |  |  |  |  |  |  |
|  |  | *r* | .26 | -.71 | - |  |  |  |  |  |  |
|  |  | *p* | .001 | <.001 | - |  |  |  |  |  |  |
|  |  | BF_10_ | 16.85 | >100 | - |  |  |  |  |  |  |
| **Spelling** | Custom task | 2a. Spelling Acc |  |  |  |  |  |  |  |  |  |
|  |  | *r* | .44 | -.18 | .33 | - |  |  |  |  |  |
|  |  | *p* | <.001 | .03 | <.001 | - |  |  |  |  |  |
|  |  | BF_10_ | >100 | 1.05 | >100 | - |  |  |  |  |  |
|  |  | 2b. Spelling RT |  |  |  |  |  |  |  |  |  |
|  |  | *r* | .22 | .52 | -.33 | -.04 | - |  |  |  |  |
|  |  | *p* | .005 | <.001 | <.001 | .60 | - |  |  |  |  |
|  |  | BF_10_ | 3.49 | >100 | >100 | 0.12 | - |  |  |  |  |
|  | Standardized task | 2c. Dictation |  |  |  |  |  |  |  |  |  |
|  |  | *r* | .33 | -.29 | .39 | .75 | -.24 | - |  |  |  |
|  |  | *p* | <.001 | <.001 | <.001 | <.001 | .004 | - |  |  |  |
|  |  | BF_10_ | >100 | 42.30 | >100 | >100 | 6.45 | - |  |  |  |
| **Metacognitive monitoring** | Calibration of confidence | 3a. MM_arith_ |  |  |  |  |  |  |  |  |  |
|  |  | *r* | .89 | .00 | .40 | .47 | .15 | .37 | - |  |  |
|  |  | *p* | <.001 | .99 | <.001 | <.001 | .08 | <.001 | - |  |  |
|  |  | BF_10_ | >100 | 0.10 | >100 | >100 | 0.46 | >100 | - |  |  |
|  |  | 3b. MM_spell_ |  |  |  |  |  |  |  |  |  |
|  |  | *r* | .52 | -.18 | .37 | .92 | -.05 | .73 | .58 | - |  |
|  |  | *p* | <.001 | .03 | <.001 | <.001 | .56 | <.001 | <.001 | - |  |
|  |  | BF_10_ | >100 | 1.14 | >100 | >100 | 0.12 | >100 | >100 | - |  |
| **Control Variables** | Intellectual ability | 4. Raven |  |  |  |  |  |  |  |  |  |
|  |  | *r* | .19 | -.02 | .16 | .34 | -.01 | .28 | .25 | .31 | - |
|  |  | *p* | .03 | .81 | .05 | <.001 | .91 | <.001 | .003 | <.001 | - |
|  |  | BF_10_ | 1.20 | 0.11 | 0.66 | >100 | 0.11 | 29.26 | 8.22 | >100 | - |
|  | Motor speed | 5. Motor speed task RT |  |  |  |  |  |  |  |  |  |
|  |  | *r* | .20 | .47 | -.32 | .00 | .33 | -.08 | .13 | .01 | -.08 |
|  |  | *p* | .02 | <.001 | <.001 | .99 | <.001 | .33 | .13 | .94 | .35 |
|  |  | BF_10_ | 1.59 | >100 | >100 | 0.10 | >100 | 0.17 | 0.33 | 0.10 | 0.16 |

Acc = accuracy; RT = response time for the correct answers.

**Table B2. Correlational analyses between the administered measures in 7-8-year-olds (Grade 2; Study 2)**

|  |  |  | 1a | 1b | 1c | 2a | 2b | 2c | 3a | 3b | 4 |
| --- | --- | --- | --- | --- | --- | --- | --- | --- | --- | --- | --- |
| **Arithmetic** | Custom task | 1a. Arithmetic ACC | - |  |  |  |  |  |  |  |  |
|  |  | 1b. Arithmetic RT |  |  |  |  |  |  |  |  |  |
|  |  | *r* | .46 | - |  |  |  |  |  |  |  |
|  |  | *p* | <.001 | - |  |  |  |  |  |  |  |
|  |  | BF_10_ | >100 | - |  |  |  |  |  |  |  |
|  | Standard task | 1c. TTA |  |  |  |  |  |  |  |  |  |
|  |  | *r* | .45 | -.05 | - |  |  |  |  |  |  |
|  |  | *p* | <.001 | .70 | - |  |  |  |  |  |  |
|  |  | BF_10_ | >100 | 0.17 | - |  |  |  |  |  |  |
| **Spelling** | Custom task | 2a. Spelling ACC |  |  |  |  |  |  |  |  |  |
|  |  | *r* | .15 | .05 | .13 | - |  |  |  |  |  |
|  |  | *p* | .22 | .70 | .28 | - |  |  |  |  |  |
|  |  | BF_10_ | 0.31 | 0.16 | 0.27 | - |  |  |  |  |  |
|  |  | 2b. Spelling RT |  |  |  |  |  |  |  |  |  |
|  |  | *r* | .01 | .33 | -.20 | -.06 | - |  |  |  |  |
|  |  | *p* | .92 | .004 | .11 | .62 | - |  |  |  |  |
|  |  | BF_10_ | 0.15 | 7.75 | 0.55 | .16 | - |  |  |  |  |
|  | Standard task | 2c. Dictation |  |  |  |  |  |  |  |  |  |
|  |  | *r* | .28 | .20 | .24 | .28 | -.05 | - |  |  |  |
|  |  | *p* | .02 | .11 | .05 | .02 | .69 | - |  |  |  |
|  |  | BF_10_ | 1.91 | 0.55 | 0.96 | 2.16 | .17 | - |  |  |  |
| **Metacognitive monitoring** | Calibration of confidence | 3a. MM_arith_ |  |  |  |  |  |  |  |  |  |
|  |  | *r* | .80 | .37 | .47 | .19 | .09 | .28 | - |  |  |
|  |  | *p* | <.001 | .001 | <.001 | .11 | .46 | .02 | - |  |  |
|  |  | BF_10_ | >100 | 20.90 | >100 | 0.53 | 0.19 | 1.87 | - |  |  |
|  |  | 3b. MM_spell_ |  |  |  |  |  |  |  |  |  |
|  |  | *r* | .18 | .19 | .08 | .90 | .02 | .36 | .26 | - |  |
|  |  | *p* | .12 | .11 | .51 | <.001 | .84 | .003 | .03 | - |  |
|  |  | BF_10_ | 0.48 | 0.52 | 0.19 | >100 | 0.15 | 13.07 | 1.64 | - |  |
| **Control Variables** | Intellectual ability | 4. Raven |  |  |  |  |  |  |  |  |  |
|  |  | *r* | .46 | .27 | .12 | .21 | -.05 | .24 | .44 | .28 | - |
|  |  | *p* | <.001 | .03 | .33 | .10 | .67 | .05 | <.001 | .02 | - |
|  |  | BF_10_ | >100 | 1.49 | 0.24 | 0.60 | 0.17 | 1.06 | 131.5 | 2.06 | - |
|  | Motor speed | 5. Motor speed task RT |  |  |  |  |  |  |  |  |  |
|  |  | *r* | .02 | .25 | -.43 | -.17 | .35 | -.13 | .05 | -.13 | -.31 |
|  |  | *p* | .87 | .04 | <.001 | .14 | .002 | .29 | .69 | .26 | .01 |
|  |  | BF_10_ | 0.15 | 1.16 | 73.64 | 0.42 | 15.17 | 0.27 | 0.16 | 0.27 | 3.32 |

ACC = accuracy; RT = response time for the correct answers.
