# Supplementary material for: Metacognition across domains: Is the association between arithmetic and metacognitive monitoring domain-specific?
Source: PLoS One. 2020 Mar 12;15(3):e0229932. doi: 10.1371/journal.pone.0229932 (PMC7067420; doi:10.1371/journal.pone.0229932)
Supplement: S3 Appendix — (DOCX) [file pone.0229932.s003.docx]

# S3 Appendix C.

## Analyses with chronological age.

Although not originally pre-registered, we additionally calculated the analyses presented in the manuscript with chronological age as an additional control variable. Pearson correlation coefficients were calculated between age and academic and metacognitive performance measures in both grades (see table C1 below). The associations between age and the other metrics were not statistically significant, and Bayes factors were all below 0.43, consequently pointing to evidence for the null hypotheses of no association between age and the variables under investigation. In line with the lack of significant correlations with age, post-hoc defined partial correlations and regression models to control for shared variance across age and the other metrics (see Tables C2-C6) indicate that including age in the analyses does not change the interpretation of the current results.

**Table C1. Correlation analyses of age and academic and metacognitive performance measures in both grades.**

|  | Study 1 - Grade 3 | Study 2 - Grade 2 |
| --- | --- | --- |
|  | Age | Age |
|  |  |  |
| Arithmetic performance |  |  |
| *Custom task* |  |  |
| Accuracy |  |  |
| *r* | -.07 | .15 |
| *p* | .44 | .22 |
| BF_10_ | 0.14 | 0.31 |
| Response time |  |  |
| *r* | -.09 | -.09 |
| *p* | .28 | .46 |
| BF_10_ | 0.19 | 0.19 |
| *Standardized task* |  |  |
| *r* | .06 | .18 |
| *p* | .46 | .15 |
| BF_10_ | 0.13 | 0.42 |
| Spelling performance |  |  |
| *Custom task* |  |  |
| Accuracy | .07 | -.14 |
| *r* | .43 | .26 |
| *p* | 0.15 | 0.28 |
| BF_10_ |  |  |
| Response time |  |  |
| *r* | -.13 | -.13 |
| *p* | .12 | .29 |
| BF_10_ | 0.36 | 0.25 |
| *Standardized task* |  |  |
| *r* | .06 | .01 |
| *p* | .46 | .94 |
| BF_10_ | 0.14 | 0.15 |
| Metacognitive Monitoring |  |  |
| *Arithmetic* |  |  |
| *r* | -.14 | .07 |
| *p* | .11 | .59 |
| BF_10_ | 0.37 | 0.17 |
| *Spelling* |  |  |
| *r* | -.01 | -.13 |
| *p* | .87 | .28 |
| BF_10_ | 0.11 | 0.26 |

**Table C2. Partial correlations of metacognitive monitoring and academic performance measures in 8-9-year-olds (Grade 3).**

|  | Arithmetic | | | Spelling | | |
| --- | --- | --- | --- | --- | --- | --- |
|  | Custom task – Accuracy^a^ | Custom task - RT ^b^ | Standardized task (TTA) ^a^ | Custom task - Accuracy^a^ | Custom task -RT ^b^ | Standardized task (dictation) ^a^ |
| Metacognitive Monitoring |  |  |  |  |  |  |
| *Arithmetic* |  |  |  |  |  |  |
| *r* | .86 | -.05 | .43 | .53 | .11 | .35 |
| *p* | <.001 | .53 | <.001 | <.001 | .22 | <.001 |
| BF_10_ | >100 | 0.13 | >100 | >100 | 0.23 | >100 |
| *Spelling* |  |  |  |  |  |  |
| *r* | .53 | -.15 | .38 | .93 | -.04 | .71 |
| *p* | <.001 | .09 | <.001 | <.001 | .68 | <.001 |
| BF_10_ | >100 | 0.45 | >100 | >100 | 0.12 | >100 |

Note. All correlations are additionally controlled for age.

^a^ Controlled for intellectual ability.

^b^ Controlled for intellectual ability and motor speed on the keyboard.

**Table C3. Partial correlations of metacognitive monitoring and academic performance measures in 7-8-year-olds (Grade 2).**

|  | Arithmetic | | | Spelling | | |
| --- | --- | --- | --- | --- | --- | --- |
|  | Custom task – Accuracy^a^ | Custom task - RT ^b^ | Standardized task (TTA) ^a^ | Custom task - Accuracy^a^ | Custom task -RT ^b^ | Standardized task (dictation) ^a^ |
| Metacognitive Monitoring |  |  |  |  |  |  |
| *Arithmetic* |  |  |  |  |  |  |
| *r* | .80 | .37 | .46 | .16 | .08 | .17 |
| *p* | <.001 | .001 | <.001 | .23 | .52 | .18 |
| BF_10_ | >100 | 20.31 | >100 | 0.32 | 0.20 | 0.38 |
| *Spelling* |  |  |  |  |  |  |
| *r* | .06 | .11 | .11 | .89 | -.01 | .36 |
| *p* | .66 | .42 | .39 | <.001 | .92 | .003 |
| BF_10_ | 0.17 | 0.22 | 0.22 | >100 | 0.16 | 11.93 |

Note. All correlations are additionally controlled for age.

^a^ Controlled for intellectual ability.

^b^ Controlled for intellectual ability and motor speed on the keyboard.

**Table C4. Partial correlations of metacognitive monitoring measures.**

|  | Study 1 – Grade 3 | Study 2 – Grade 2 |
| --- | --- | --- |
|  | Metacognitive monitoring Spelling | Metacognitive monitoring Spelling |
| Metacognitive monitoring Arithmetic |  |  |
| *r* | .41 ^a^ | .17 ^b^ |
| *p* | <.001 | .19 |
| BF_10_ | >100 | 0.37 |

Note. ^a^ Partial correlation controlled for intellectual ability, arithmetic and spelling performance on the standardized tasks and age; ^b^ Partial correlation controlled for intellectual ability and age.

**Table C5. Regression analyses of MM_arith_ and MM_spell_ performance with metacognitive monitoring in the other domain, standardized task performance in both domains and age as predictors (Grade 3).**

|  | MM_arith_ | | | |
| --- | --- | --- | --- | --- |
|  | *β* | *t* | *p* | BF_inclusion_ |
| Age | -.12 | -1.77 | .08 | 2.04 |
| Intellectual ability | .14 | 1.91 | .06 | 2.12 |
| TTA | .27 | 3.68 | <.001 | 84.62 |
| Dictation | -.12 | -1.18 | .24 | 1.06 |
| MM_spell_ | .49 | 4.93 | <.001 | >100 |
|  | MM_spell_ | | | |
|  | *β* | *t* | *p* | BF_inclusion_ |
| Age | .01 | .21 | .84 | 0.19 |
| Intellectual ability | .08 | 1.28 | .20 | 0.36 |
| Dictation | .55 | 8.49 | <.001 | >100 |
| TTA | -.001 | -.01 | .99 | 0.19 |
| MM_arith_ | .34 | 4.93 | <.001 | >100 |

**Table C6. Regression analyses of arithmetic performance (i.e., arithmetic_acc_ and TTA) and spelling performance (i.e., spelling_acc_ and dictation) with metacognitive monitoring in the other domain, standardized task performance in the other domain and age as predictors (Grade 3).**

|  | Arithmetic | | | | | | | |
| --- | --- | --- | --- | --- | --- | --- | --- | --- |
|  | Arithmetic_acc_ | | | | TTA | | | |
|  | *β* | *t* | *p* | BF_inclusion_ | *β* | *t* | *p* | BF_inclusion_ |
| Age | -.04 | -.52 | .61 | .29 | .04 | .48 | .63 | 0.42 |
| MM_spell_ | .53 | 4.99 | <.001 | >100 | .22 | 1.95 | .05 | 3.19 |
| Dictation | -.07 | -.64 | .53 | .30 | .19 | 1.68 | .10 | 1.83 |
|  | Spelling | | | | | | | |
|  | Spelling_acc_ | | | | Dictation | | | |
|  | *β* | *t* | *p* | BF_inclusion_ | *β* | *t* | *p* | BF_inclusion_ |
| Age | .13 | 1.75 | .08 | 1.24 | .10 | 1.18 | .24 | 1.14 |
| MM_arith_ | .50 | 6.06 | <.001 | >100 | .25 | 2.80 | .006 | 10.16 |
| TTA | .09 | 1.15 | .25 | 0.68 | .23 | 2.63 | .01 | 11.67 |
