# Supplementary material for: Metacognition across domains: Is the association between arithmetic and metacognitive monitoring domain-specific?
Source: PLoS One. 2020 Mar 12;15(3):e0229932. doi: 10.1371/journal.pone.0229932 (PMC7067420; doi:10.1371/journal.pone.0229932)
Supplement: S1 File — (DOCX) [file pone.0229932.s004.docx]

**S4 Supplementary materials.**

In these supplementary materials, all remaining preregistered analyses for both studies that were not presented in the main body of the manuscript are presented.

# Study 1

## Preliminary correlational analyses.

As a validity check, we first verified whether the performance measures of the custom tasks that we made for this study were correlated with the widely-used standardized arithmetic and spelling tasks that we utilized (i.e., TTA and dictation). This was the case for both the accuracy (arithmetic: *r* = .257, *p* = .002; spelling: *r* = .748, *p* < .001) and the response time for correct answers (arithmetic: *r* = -.709, *p* < .001; spelling: *r* = -.248, *p* = .003).

## General metacognitive knowledge.

### Method

To measure metacognitive abilities independently of arithmetic and spelling, we used a general metacognitive questionnaire (adapted from Haberkorn, Lockl, Pohl, Ebert, & Weinert, 2014). In this questionnaire, 15 situations involving mental performance (e.g., *Which strategy do you think is better to make sure you won’t forget to take your skates to school the next day?*) were described and three possible answers (e.g., *a. Write a note on a piece of paper; b. Think strongly about the skates; c. Both proposed strategies are equally good/bad*) were presented. The researcher read the situations and the corresponding options aloud one by one. Children were given a response form with pictures of the three possible answers, so they could follow each item and indicate their answer. The performance measure was the number of correct answers. The mean score on the general metacognitive knowledge questionnaire was 10.70 (SD = 2.35; range [4.00-15.00]).

The preregistered analyses involving the general metacognitive knowledge questionnaire are presented below.

### Associations of academic performance and general metacognitive knowledge

Table S1

*Correlational analyses of metacognition measures and academic performance measures in 8-9-year-olds (Grade 3)*

|  | Arithmetic | | | Spelling | | |
| --- | --- | --- | --- | --- | --- | --- |
|  | Custom task – Accuracy^a^ | Custom task - RT ^b^ | Standardized task (TTA) ^a^ | Custom task - Accuracy^a^ | Custom task -RT ^b^ | Standardized task (dictation) ^a^ |
| Metacognitive knowledge |  |  |  |  |  |  |
| *r* | .14 | -.02 | .06 | .12 | .03 | .15 |
| *p* | .12 | .81 | .50 | .17 | .75 | .08 |
| BF_10_ | 0.36 | 0.11 | 0.13 | 0.27 | 0.11 | 0.49 |

*Note.* ^a^ Controlled for intellectual ability; ^b^ Controlled for intellectual ability and motor speed on the keyboard.

The general metacognitive knowledge questionnaire (MC_know_) was not significantly associated with the arithmetic and spelling measures. For the arithmetic measures, the Bayes factors indicated moderate evidence in favour of no association with Arithmetic_rt_ and the TTA; there was no considerable evidence in favour of or against an association with Arithmetic_acc_. For the spelling measures, the Bayes factors indicated moderate evidence in favour of no association with Spelling_acc_ and Spelling_rt_; there was no considerable evidence in favour of or against an association with dictation.

### The unique role in academic performance of metacognitive monitoring within- and across-domain and metacognitive knowledge.

The correlational analyses show significant associations between our academic performance measures and metacognitive monitoring within the domain, in contrast to no significant correlations and (moderate) evidence in favour of no associations of the academic performance measures and general metacognitive knowledge. Comparing the strength of the associations confirms this pattern: For every performance measure, the strength of the association with metacognitive monitoring within the domain was significantly larger than the strength of the association with general metacognitive knowledge (William-Steiger tests; all *p*’s < .001).

Regression analyses were performed to assess the unique contribution of our different metacognitive measures (i.e., metacognitive monitoring within- and cross-domain and general metacognitive knowledge) to arithmetic and spelling performance. Therefore, all metacognitive measures were simultaneously entered into the regression models, together with intellectual ability as a control measure (Table S2). For every model, all variance inflation factors (VIF) were smaller than 1.60, indicating no issues with collinearity among predictors.

Table S2

*Regression analyses of arithmetic and spelling performance with metacognitive monitoring within- and cross-domain, MC_know_ and intellectual ability as predictors*

|  | Arithmetic | | | | | | | | Spelling | | | | | | | |
| --- | --- | --- | --- | --- | --- | --- | --- | --- | --- | --- | --- | --- | --- | --- | --- | --- |
|  | Arithmetic_acc_ | | | | Standardized task (TTA) | | | | Spelling_acc_ | | | | Standardized task (dictation) | | | |
|  | *β* | *t* | *p* | BF _inclusion_ | *β* | *t* | *p* | BF _inclusion_ | *β* | *t* | *p* | BF _inclusion_ | *β* | *t* | *p* | BF _inclusion_ |
| MM_arith_ | .83 | 15.06 | <.001 | >100 | .33 | 3.36 | .001 | 97.81 | -.04 | -0.98 | .33 | 0.06 | -.05 | -0.67 | .51 | 0.18 |
| MM_spell_ | .04 | 0.69 | .50 | 0.09 | .15 | 1.52 | .13 | 0.70 | .93 | 23.11 | <.001 | >100 | .69 | 8.91 | <.001 | >100 |
| MC_know_ | .08 | 1.88 | .06 | 0.33 | -.01 | -0.17 | .87 | 0.28 | .02 | 0.76 | .45 | 0.06 | .10 | 1.63 | .11 | 0.43 |
| intellectual ability | -.01 | -0.12 | .90 | 0.07 | .02 | 0.26 | .80 | 0.30 | .04 | 1.23 | .22 | 0.08 | .09 | 1.38 | .17 | 0.30 |

These results show that when metacognitive monitoring within- and across-domain, and general metacognitive knowledge were considered simultaneously, only the role of metacognitive monitoring within the domain itself remained significant (frequentist statistics) or supported (Bayesian statistics) for each academic performance measure (i.e., Arithmetic_acc_, Spelling_acc_, TTA, dictation).

### Discussion.

Whereas the lack of a significant/supported association of academic performance with MC_know_ suggests that domain-generality of metacognition might be limited to metacognitive monitoring within the academic domains, it is important to note some characteristics of this study that might limit this interpretation of the specificity of metacognition. The current study included two aspects of metacognition, namely declarative, general metacognitive knowledge (MC_know_) and an aspect of procedural metacognition (i.e., metacognitive monitoring; MM_arith_ and MM_spell_). In all above-mentioned results, the role of metacognitive monitoring surpasses the role of general metacognitive knowledge in academic performance. Firstly, this difference in results could be due to a difference in the cognitive process, namely declarative vs. procedural metacognition. It is not surprising that different aspects of metacognition follow different developmental paths (Schneider & Löffler, 2016) and are differently associated with domain-specific skills. To further investigate whether this difference in results is indeed due to a difference in metacognitive processes, future studies should not only include domain-general declarative metacognitive knowledge, but also measures of domain-specific, declarative metacognitive knowledge. Secondly, the difference in results between metacognitive monitoring and declarative metacognitive knowledge could be due to a difference in performance measures that were used to measure these skills. The general metacognitive knowledge questionnaire (used to measure declarative, general metacognitive knowledge), while being a validated measure, differs in two considerable ways from our monitoring measure, which was online and more detailed (i.e., on a trial-by-trial basis). These aspects of the current study could limit the interpretation of the specificity of metacognition outside the academic domains and outside of metacognitive monitoring. Drawing on our results, future research should include measures of both metacognitive monitoring and control (i.e., both aspects of procedural metacognition) and general as well as domain-specific declarative metacognitive knowledge (e.g., Neuenhaus, Artelt, Lingel, & Schneider, 2011) to further investigate the domain-specificity question of metacognition.

## Academic-domain-related differences in the role of metacognitive monitoring.

We investigated whether there were academic-domain-related differences (i.e., differences between arithmetic and spelling) in the (strength of the) role of metacognition (i.e., task-specific metacognitive monitoring on the one hand and general metacognitive knowledge on the other).

Our results show that there were differences in the strength of the role of metacognitive monitoring depending on the domain. Using the Fisher r-to-z transformation, we found that the association between MM_spell_ and Spelling_acc_ was significantly larger than the association between MM_arith_ and Arithmetic_acc_ (*p* = .014). This was also the case for the standardized task, with the association between MM_spell_ and dictation being significantly larger than the association between MM_arith_ and the TTA (*p* = .001). There were no significant differences between the domains in the strength of the associations with MC_know_.

# Study 2

## Preliminary correlational analyses.

The same validity tests were performed as in Study 1, showing that for accuracy (arithmetic: *r* = .46, *p* < .001, BF_10_ > 100; spelling: *r* = .28, *p* = .02, BF_10_ = 2.16) performance on the custom task was correlated with the standardized task. For response time for correct answers (arithmetic: *r* = -.05, *p* = .70, BF_10_ = 0.17; spelling: *r* = -.05, *p* = .69, BF_10_ = 0.17) this was not the case.

## General metacognitive knowledge.

The preregistered analyses involving the general metacognitive knowledge questionnaire in Grade 2 are presented below. The mean score on the general metacognitive knowledge questionnaire was 6.65 (SD = 2.44; range [1.00-11.00]).

### Associations of academic performance and general metacognitive knowledge

Table S3

*Correlational analyses of metacognition measures and academic performance measures in 7-8-year-olds (Grade 2)*

|  | Arithmetic | | | Spelling | | |
| --- | --- | --- | --- | --- | --- | --- |
|  | Custom task – Accuracy^a^ | Custom task - RT ^b^ | Standardized task (TTA) ^a^ | Custom task - Accuracy^a^ | Custom task -RT ^b^ | Standardized task (dictation) ^a^ |
| Metacognitive knowledge |  |  |  |  |  |  |
| *r* | .21 | .05 | .23 | .10 | .03 | .05 |
| *p* | .09 | .68 | .07 | .43 | .84 | .71 |
| BF_10_ | 0.62 | 0.16 | 0.79 | 0.21 | 0.16 | 0.16 |

*Note.* ^a^ Controlled for intellectual ability; ^b^ Controlled for intellectual ability and motor speed on the keyboard.

The general metacognitive knowledge questionnaire (MC_know_) was not significantly associated with the arithmetic and spelling measures. Bayes factors indicated moderated evidence in favour of no association with both response time measures, and for Spelling_acc_ and dictation. There was only anecdotal evidence for no association with Arithmetic_acc_ and TTA.

Based on the lack of significant/supported associations, no further analyses were performed.

## Academic-domain-related differences in the role of metacognition.

We investigated whether there were academic-domain-related differences (i.e., differences between arithmetic and spelling) in the (strength of the) role of metacognition (i.e., task-specific metacognitive monitoring on the one hand and general metacognitive knowledge on the other).

Our results show that there were almost no differences in the strength of the role of metacognitive monitoring depending on the domain. Using the Fisher r-to-z transformation, we found that only the association between MM_spell_ and Spelling_acc_ was significantly larger than the association between MM_arith_ and Arithmetic_acc_ (*p* = .005). There were no other significant differences between the domains in the strength of the associations with metacognition.
