# Supplementary material for: Metacognition across domains: Is the association between arithmetic and metacognitive monitoring domain-specific?
Source: PLoS One. 2020 Mar 12;15(3):e0229932. doi: 10.1371/journal.pone.0229932 (PMC7067420; doi:10.1371/journal.pone.0229932)
Supplement: Attachment — Submitted filename: ResponsLetter_RevisedManuscript.docx [file pone.0229932.s006.docx]

Response to Reviewers - PONE-D-19-26504

Metacognition across domains: Is the association between arithmetic and metacognitive monitoring domain-specific?

Dear Editor and Reviewers,

We thank you for your careful rereading of our revised manuscript and response letter, and for your positive evaluation of our manuscript. We appreciate the time and effort that you have dedicated to reviewing our work. We have taken your comments into account in our revision. Please find below our point-by-point response to your comments and queries. To ease the identification of changes to the text in the revised manuscript, we have highlighted all changes by using coloured text.

# Responses to the Editor

## Editor, general point

Thank you for submitting your revised manuscript to PLOS ONE. I have sent your paper back to the original reviewers and have now received their comments. Both are positive about this version and recommend acceptance. I agree with them.  However, the reviewers also provide some further suggestions to clarify a few points. Therefore, I would like to give you the opportunity to address these comments in a minor revision before acceptance.

**Author’s response**: We thank the Editor and the Reviewers for their positive evaluation of the manuscript and their suggestions for some further clarifications. We agree that these were areas with room for improvement and have revised the manuscript in accordance with these comments. Specifically, we have rephrased longer sentences in our manuscript, splitting them into shorter, clearer sentences. We further addressed the confusion about the number of practice trials in the custom academic tasks. In the Supplementary materials section on the general metacognitive knowledge questionnaire, we added the descriptive statistics of this measure. Lastly, we have thoroughly checked the references to age and grade in the manuscript to ensure consistency. In the remainder of this response letter, we provide a point-by-point response to the points raised by the two Reviewers.

# Responses to Reviewer 1

## Reviewer 1, general point

I really appreciate the thorough and thoughtful responses from the authors, the additional analyses to demonstrate the robustness of findings, and the authors making the study and the findings extremely transparent! I only have three very minor comments for the authors to consider.

**Author’s response**: We thank the Reviewer for his/her thoughtful, positive feedback on our revised manuscript and response letter.

## Reviewer 1, point 1

Introduction
A very minor thing, I noticed that “Rinne & Mazzocco [6]” is referenced in text but perhaps “&” should be replaced with “and” (pages 6-7). I think “&” is usually used in parentheses citations (e.g., One recent study found (Rinne & Mazzocco, 2013)….), and “and” is usually used when the citation is a part of a sentence (e.g., Rinne and Mazzocco (2013) found…).

**Author’s response**: We thank the Reviewer for highlighting this. We have changed this on pages 6-7 as indicated by the Reviewer and additionally checked the entire manuscript for sentences which had the same issue and adjusted this accordingly, i.e. on page 19 (i.e., Arithmetic_acc_ and TTA instead of Arithmetic_acc_ & TTA).

## Reviewer 1, point 2

Study 1

It seems that there were four practice items to familiarize children with the metacognitive monitoring measure in the custom computerized tasks (page 13). However, based on the description of the custom computerized spelling task (page 11), there were six practice trials proceeded the block with 60 trials that included the metacognitive monitoring measure. Are the six practice trials in the computerized spelling task different from the four practice items described in the metacognitive monitoring section? I thought the practice items described in the spelling section (second block of computer task) and in the metacognitive monitoring section would be the same but the number of trials were different so perhaps I was wrong.

**Author’s response**: We fully agree with the Reviewer that that this wording may confuse the reader. The Reviewer is correct in assuming that the practice trials described in the introductory paragraph on the metacognitive monitoring task (page 13) are the same as those described in the individual sections on the custom tasks. In the custom spelling task, there were six practice items, in the custom arithmetic task, there were four practice items. The rationale behind the number of practice items was twofold. Firstly, in both the spelling and the arithmetic task, the number of practice items reflected the number of different task components. In the spelling task, three specific spelling rules were included in the task. The practice items thus encompassed two items per rule, yielding six items. In the arithmetic task, two operations were included (i.e., addition and multiplication). The practice items thus encompassed two items per operation, yielding four items. Secondly, the spelling task was the first task in the protocol for which children had to use the computer and in which the metacognitive protocol was implemented. To make sure children were familiar with these task requirements, the number of practice items was higher than in the arithmetic custom task, which was administered later in the protocol. Hence, when this arithmetic task was administered, children already had some experience with the general task requirements.

To prevent confusion about the number of practice items, we have revised the reference to practice items in the introductory paragraph on the metacognitive monitoring task (page 13), removing the reference to the number of trials. This informs the reader that practice items were administered, and the specific number of practice items for each task is then presented under its task description.

As a result, the manuscript includes the following references to the practice items:

- Manuscript page 13 (introductory paragraph on metacognitive monitoring task):

“*To familiarize the children with the task, in each task practice items were presented.”*

- Manuscript page … (paragraph on metacognitive monitoring in the arithmetic task):

*“Each block was preceded by four practice trials to familiarize the child with the task requirements.”*

- Manuscript page 11 (paragraph on metacognitive monitoring in the spelling task):

“*Each block was preceded by six practice trials to familiarize the child with the task requirements*.”

## Reviewer 1, point 3

S4 Supplementary Materials

General metacognitive knowledge seems to have no association with arithmetic and spelling performances at all in study 1(page 2) and study 2 (page 6). I wonder whether children scored very high on this questionnaire, and their scores restricted the range for correlations. Perhaps it would be good to report means and standard deviations of general metacognitive knowledge for both age groups.

**Author’s response**: We completely agree with the Reviewer that reporting means and standard deviations of the general metacognitive knowledge questionnaire would improve the transparency of the findings on this task. We have added these for both age groups in the Supplementary material (i.e., S4). This resulted in the following addition to the supplementary materials:

S4 page 1 (i.e., Results Study 1): “*The mean score on the general metacognitive knowledge questionnaire was 10.70 (SD = 2.35; range [4.00-15.00]).*”

S4 page 6 (i.e., Results Study 2): “*The mean score on the general metacognitive knowledge questionnaire was 6.65 (SD = 2.44; range [1.00-11.00]).”*

## Reviewer 1, point 4

The data are not yet available. The authors indicated that the data set will be available after manuscript publication

**Author’s response**: Indeed, we will make the data available through the Open Science Framework page of the current project after manuscript publication (<https://osf.io/ypue4/?view_only=ce9f97af0e3149c28942a43499eafd32>) .

# Responses to Reviewer 2

## Reviewer 2, point 1

I am recommending acceptance as the authors have done a nice job addressing my prior concerns. However, I just wanted to point out a minor thing. This is not necessarily a critique but hopefully a helpful suggestion to ensure novice readers don't get tripped up on the opening paragraph. The authors edited the second sentence of the paper to address a prior concern. By doing so, they have inadvertently made it quite long and difficult to parse. Given that this sentence introduces the main topic, it might be worth breaking it up into several key ideas. For example, the first key idea is that "learning from mistakes is a facet of metacognition." The second key idea is the definition of metacognition. And the third main point is the definitions of declarative vs. procedural metacognition. I would suggest using three brief sentences so that it's easier to follow. Also, I still found it a bit confusing that "declarative" metacognition is defined as "the ability to assess" cognition and metacognitive monitoring is also defined as an ability to make "self-assessments." Since monitoring is a part of procedural metacognition, it's confusing that its definition is so similar to declarative metacognition. Because this paper does not deal with declarative metacognition, my suggestion is to cut this reference to it and just focus on the procedural aspect. "One critical component of metacognition is procedural metacognition, which is the..." There are a few other long sentences in the paper that seem to contain multiple big ideas that might be worth editing. Also, throughout the paper I would suggest trying to be consistent as to whether the authors refer to the children's ages or grades. For the most part, the authors refer to ages, but occasionally only refer to them by grade level. Consistency is helpful.

I look forward to seeing the published version.

**Author’s response**: We thank the Reviewer for this constructive suggestion. We fully agree that due to the revision, this sentence has become quite long and difficult. We have altered the sentence as was suggested by the Reviewer, using shorter sentences instead of one long sentence (see below).

We further agree with the Reviewer that the reference to declarative metacognition is not necessary for the current manuscript, as it does not deal with declarative metacognition. As such, it might cause confusion among the readers. As the Reviewer suggested, we have removed this reference to declarative metacognition, and adapted the sentence according to the Reviewers suggestion. This resulted in the following revised sentences in the manuscript on page 3:

*“Learn from your mistakes” is an old saying that (grand)parents teach their children. This goes back to the premise that making mistakes is associated with learning. Noticing your mistakes is an example of monitoring your cognition. This monitoring of cognition is a facet of metacognition, a concept first introduced by Flavell [1]. One critical component of metacognition is procedural metacognition. This is a self-reflecting, higher-order cognitive process, which indicates how people monitor and control their cognition during ongoing cognitive processes [2,3]. Metacognitive monitoring is an important aspect of procedural metacognition and is defined as the subjective self-assessment of how well a cognitive task will be/is/has been performed [3,4].”*

In addition to having revised the wording of the sentences pointed to by the Reviewer in the introduction, we have checked the entire manuscript for sentences that may be unnecessary long and/or containing multiple big ideas. We have adjusted the wording of these sentences to shorten them, without altering the content/message in them.

Lastly, we have checked the entire manuscript to ensure consistency in the reference to age and grade. Throughout the manuscript, we consistently used age, with additional reference (e.g., within brackets) to grade to make the distinction between the two samples as clear as possible. The reference to grade was maintained within the manuscript, because the educational grade of the children was important for the current findings, which indicated differences in results between grades, yet no association with chronological age. When specifically focussing on age within grade, we have used the term “chronological age within grade” to limit possible confusion among the readers.

REFERENCES

1.	Flavell JH. Metacognition and cognitive monitoring: A new area of cognitive-developmental inquiry. Am Psychol. 1979;34(10):906–11.

2.	Flavell JH. Cognitive development: Children’s knowledge about the mind. Annu Rev Psychol. 1999;50:21–45.

3.	Nelson TO, Narens L. Metamemory: A theoretical framework and new findings. Psychol Learn Motiv. 1990;26:125–73.

4.	Morsanyi K, Cheallaigh NN, Ackerman R. Mathematics anxiety and metacognitive processes: Proposal for a new line of inquiry. Psychol Top. 2019;28(1):147–69.
